# The reform study: a case study of embedded trials

**DOI:** 10.1186/1745-6215-16-S2-P174

**Published:** 2015-11-16

**Authors:** Sarah Cockayne, Joy Adamson, Belen Corbacho, Caroline Fairhurst, Lisa Farndon, Kate Hicks, Anne-Maree Keenan, Sally Lamb, Lorraine Loughrey, Caroline McIntosh, Hylton Menz, Anthony Redmond, Sara Rodgers, Wesley Vernon, Jude Watson, David Torgerson

**Affiliations:** 1 University of York, York, UK; 2 Sheffield Teaching Hospitals, Sheffield, UK; 3 NIHR Leeds Musculoskeletal Biomedical Research Unit, Leeds, UK; 4 Leeds Institute of Rheumatic and Musculoskeletal Medicine, Leeds, UK; 5 University of Oxford, Oxford, UK; 6 NUI Galway, Galway, Ireland; 7 La Trobe University, Bundoora, Australia

## 

Evaluation of interventions to enhance recruitment or reduce attrition within randomised controlled trials is uncommon. A number of initiatives have tried to increase this evidence base by encouraging the embedding of such trials within trials evaluating healthcare interventions.

The NIHR-funded REFORM study recruited and followed up participants by mailing out invitation packs and questionnaires to participants. We undertook four embedded studies during the recruitment and follow-up phases: (1) Exploration of the feasibility and validity of the EQ5D-5L: 332 participants were sent a baseline questionnaire containing both the EQ5D-5L and the EQ5D-3L; (2) Two embedded trials evaluating i) the effectiveness of an enhanced patient information sheet (PIS) and ii) pre-notification with a study newsletter, to increase recruitment to the trial; and (3) An embedded factorial trial evaluating the effectiveness of a Post-it® note and/or newsletter to increase questionnaire response rates and minimise attrition to the trial.

The EQ5D-5L and Post-It® note studies were easily incorporated using in-house funding. The PIS study required £6,500 funding from the MRC START team. Undertaking these studies did cause some delay to the main study, but not to the detriment of the study. To date, results for only the PIS and pre-notification studies are available, and no statistically significant differences have been observed.

We have demonstrated that it can be relatively easy to embed several trials within a trial. Whilst funding in some cases may be an issue, can provide useful learning experiences for inexperienced researchers and inform future studies and they do produce academic publications.

